# Isolation of bioactive compounds from low-cost agricultural resources and its utilization in daily life

**DOI:** 10.1099/acmi.0.000660.v4

**Published:** 2024-06-26

**Authors:** Anirban Debnath, Arpita Das

**Affiliations:** 1 Department of Biotechnology, Adamas University, Barasat, Kolkata, West Bengal 700126, India

**Keywords:** antimicrobial, bioactive compound, low-cost agricultural resources, plant extract, radical Scavenging activity

## Abstract

The ethanolic (80 %), methanolic (80 %) and aqueous decoction (100 % distilled water) of whole plant of *Oxalis corniculata* Linn (Indian Sorrel) was evaluated for its anti‐microbial and antioxidant properties by *in vitro* methods. Methanolic (80 %) and ethanolic (80 %) decoctions showed significant antibacterial property against *Staphylococcus aureus*, *Bacillus subtilis*, *Escherichia coli,* and *Salmonella typhi* bacterial strains. In comparison to Chloramphenicol (C30) against bacteria, 80 % ethanolic decoctions showed significant effect, among the decoctions. Nowadays though the standard soap is in huge demand but it’s also facing major backlash due to the presence of synthetic compounds in it, which over long use may cause harmful effects on the skin health. Therefore, the organic soaps which are made up of natural ingredients, herbs or any sort Ayurvedic compound have fewer side effects on the human skin and are much safer than standard daily soap. The formulated therapeutic soap exhibits a significant amount of reducing potential (high FRAP and TAC values) and antioxidant activity (DPPH, ABTS assay).

## Data Summary

One-way ANOVA was performed for all antioxidant, scavenging and anti-microbial assays with the help of GraphPad Prism software. RSM was performed to visualize the interaction effects of the variables using Design Expert.

## Introduction

Bioactive compounds are referred to as the extra nutritional food components present in small amounts in plant-based foods that governs diverse health benefits [1]. In plants, the bioactive compounds are mainly produced in the form of secondary metabolites [2]. Various disorders are treated locally with Indian Sorrel. It is used as an antibacterial, cooling agent, niacin, vitamin C, and β-carotene. It also functions as an adjunctive therapy for the treatment of wounds [3,5]. Avoiding the use of risky synthetic chemicals in medical soap products is preferred due to the negative or hazardous consequences of these chemicals. Recent years have seen a rise in the use of plant-based natural products as a synthetic ingredient to enhance the essential biological qualities of medicinal soap [6,9]. One of the main medicinal plants that originated in the tropics and subtropics is Indian Sorrel. It can used as a traditional medicine in systems of traditional medicine like Ayurveda, Unani, and Siddha [10,13]. Oxalidaceae can cure liver problems, skin conditions, and urinary tract issues in conventional medicine. Indian Sorrel contains fatty acids, including stearic, palmitic, oleic, and linolenic acids, all of which have different pharmacological effects [14,16]. Vitamin E, which has biological action as an antioxidant, is present in the methanol extract of *O. corniculata* [17,19]. The entire *O. corniculata* has been used to isolate the sitosterol components betulin, p-hydroxybenzoic acid, ethyl gallate, methoxy flavones, etc. [20,23]. Due to lack of proper knowledge and experimental evidence a huge amount of agricultural resources are mismanaged in the form of agro wastes despite possessing beneficial health effects [24,25].

In our present study, the scavenging activity and antimicrobial efficiency of leaves of *Oxalis corniculata* Linn. was evaluated, also a therapeutic soap formulation was created.

## Methods

### Plant extract preparation and therapeutic soap formulation

**Fig. 1.**
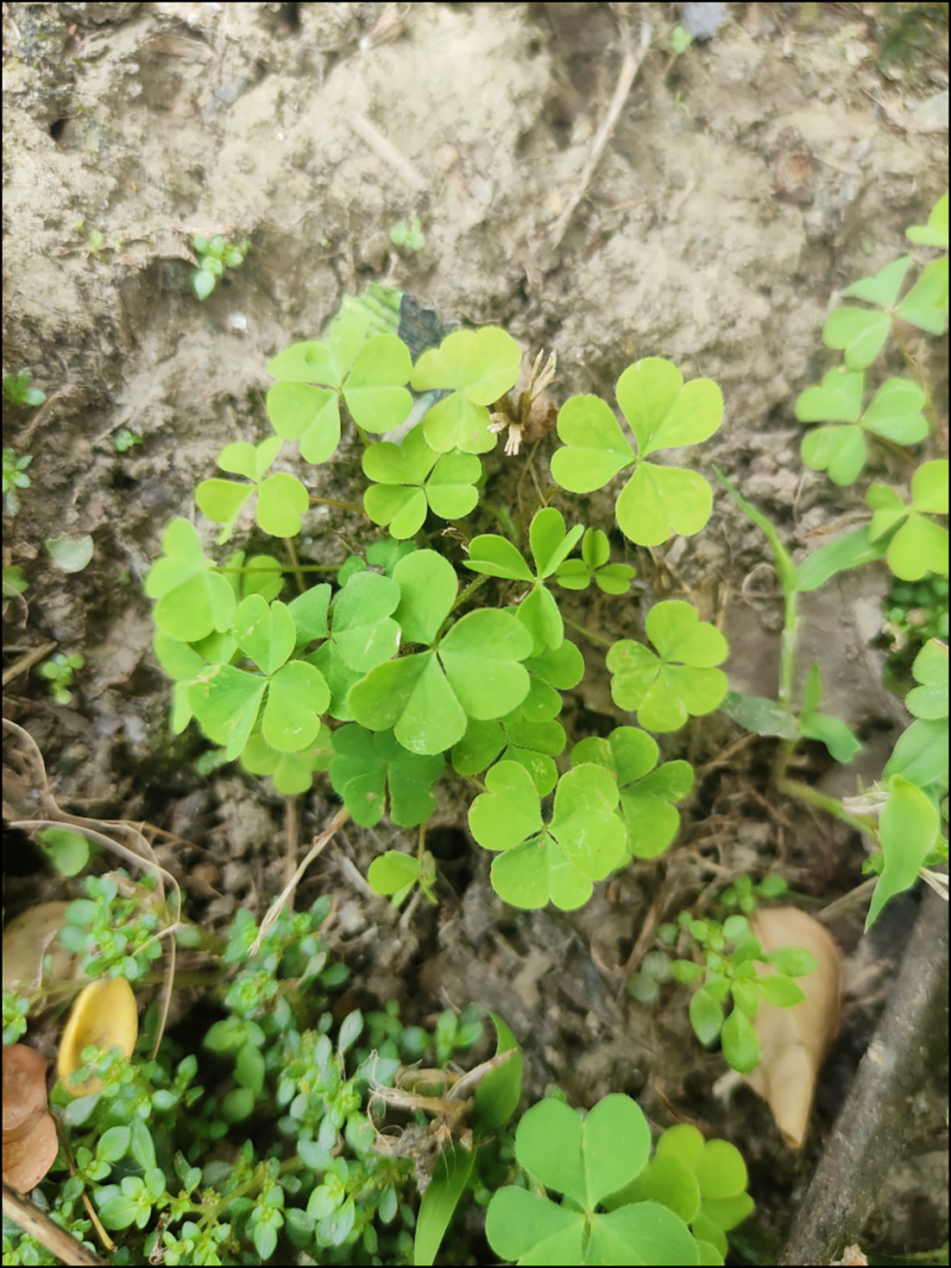
*Oxalis corniculata* Linn.

The whole plant of *Oxalis corniculata* Linn. was obtained from the campus of Adamas University located in Barasat, West Bengal, India (Coordinates 22.740868681325516, 88.45751085452098) as shown in Fig. 1. The plant Indian Sorrel (*Oxalis corniculata* Linn) were authenticated by the taxonomist of the Botanical Survey of India, Kolkata (CNH/Tech.II/2024/24). Samples were allowed to dry at 30 °C for 24 h followed by homogenizing using a mortar and pestle to prepare the extract. Two grams of powdered plant (*Oxalis corniculata* Linn) was used for extraction using 10 ml of three different solvents including 80 % ethanol (Et), 80 % methanol (Mt) and 100 % distilled water (dH_2_O) respectively. The extracts were dried to powder using a rotary vacuum evaporator (Superfit ROTAVAP PBU-6). Ten percent w/v solutions of each of powder were used as samples. The necessary chemicals were procured from different reputed companies like Folin-Ciocalteu reagent (P20571), ascorbic acid (A33117) was purchased from NICE, DPPH (29128) and ABTS (28042) were obtained from SRL.

**Fig. 2.**
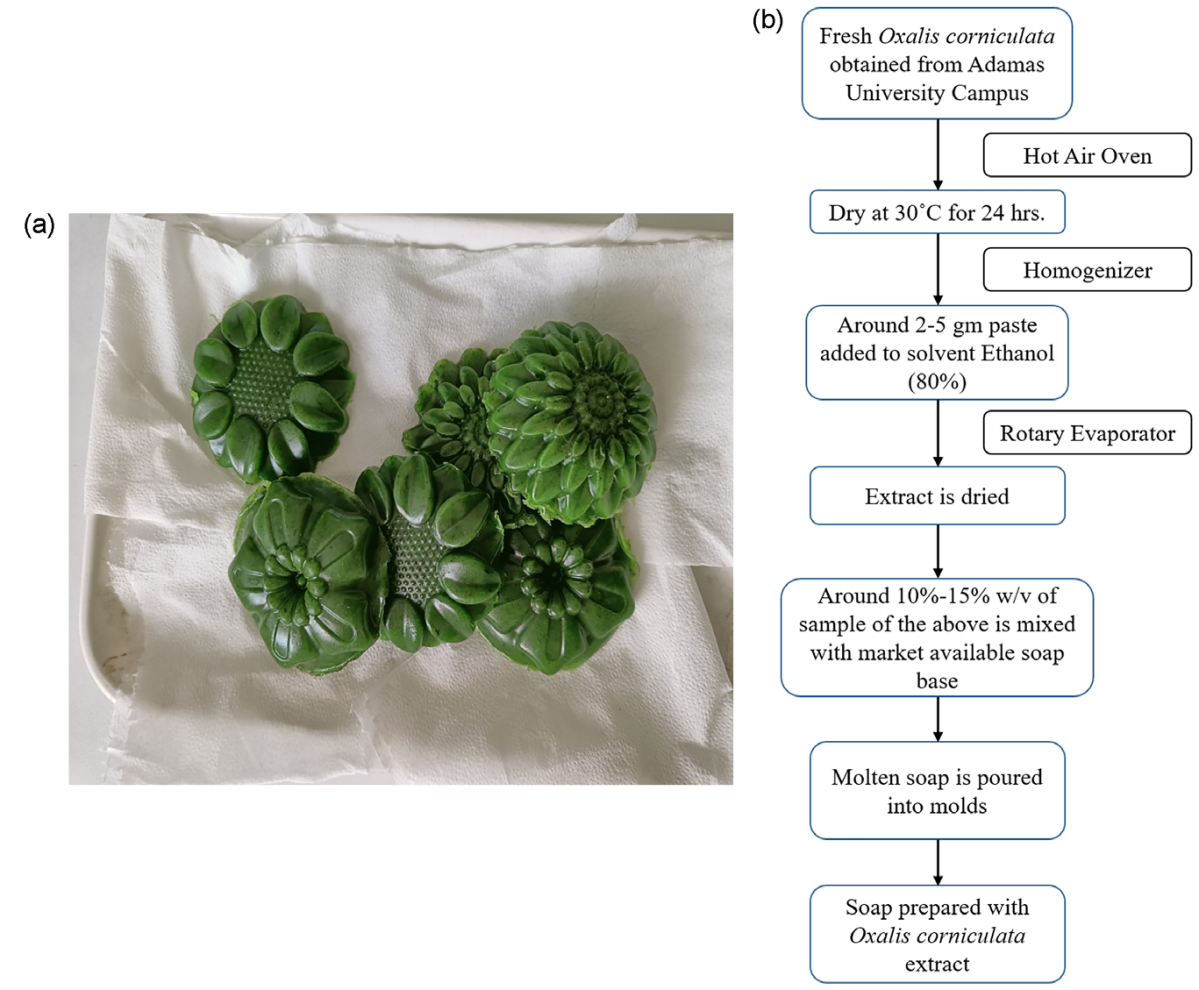
(a) Soap made with *Oxalis corniculata* Linn extract. (b) Flow chart representing the steps of herbal soap formulation from *Oxalis corniculata* Linn leaf extract.

The market available soap base was heated in the microwave for melting and collected in a beaker. Freshly prepared dried powder 10 % w/v of ethanolic extract of *O. corniculata* Linn. was poured onto the mould and allowed to cool for 24 h. The mould was removed after the soap was formed as shown in Fig. 2a. The whole experimental design from plant extract preparation to soap formulation is depicted in a flowchart below (Fig. 2b).

### Determination of phenol content and flavonoid content

The total phenolic content (TPC) of *Oxalis corniculata* Linn. plant extract in different solvents and formulated soap were determined with the help of colorimetric Folin-Ciocalteu reagent using gallic acid as standard. A reaction mixture containing 100 µl of plant extract, 1000 µl of both Folin-Ciocalteu reagent (diluted 20 times) and sodium carbonate (7 %) was incubated in the dark for 1 h 30 min and absorbance at 760 nm was taken with a spectrophotometer (Hitachi, U-2910) and the results were reported as gallic acid equivalent in milligrams GAE per gram Dry Weight (DW).

The total flavonoid content (TFC) of *Oxalis corniculata* Linn. in different solvent extracts and formulated soap were measured by a spectrophotometric method. Ten millilitres of total reaction mix including 1 ml of sample extract and 4 ml of distilled water was incubated for 5 min and mixed with 0.3 ml of 5 % sodium nitrite and 0.3 ml of 10 % aluminium chloride to incubate for 6 mins. Then 1 ml sodium hydroxide (1 M) was poured into the reaction mix, making a final volume of 10 ml. After 1 min, the absorbance was measured at 510 nm and results were shown as Quercetin equivalent (QE) of Dry Weight (DW) [26,28].

### Reducing power assay and total antioxidant assay

Measurement of antioxidant potential of *Oxalis corniculata* Linn. plant extract in different solvents and formulated soap were carried out by estimating the reduction of ferric iron to ferrous iron (ferric reducing power/ FRAP assay). A phosphomolybdenum method was also carried out to evaluate total antioxidant capacity (TAC) of *Oxalis corniculata* Linn. in different solvent extracts and formulated soap [29,31].

### ABTS assay

The antioxidant potential of *Oxalis corniculata* Linn. in different solvent extracts and formulated soap were determined by ABTS free radical scavenging assay. Briefly, two solutions of 7 mM 2,2′-azinobis-(3-ethylbenzothiaziline-6-sulfonate) and 2.45 mM of potassium persulfate were mixed in 1 : 1 ratio and kept in the dark up to 24–48 h. Two millilitres of ABTS•+ solution diluted with aqueous methanol in 1 : 25 ratio was added in an aliquot of ten times diluted 20 µl plant extract and incubated at 30 °C followed by reading the absorbance at 734 nm after 0, 10, and 20 min where ascorbic acid serves as standard [32,34].

$$\text{ABTS}\;(\%)=[({\text{A}}_{\text{Control}}-{\text{A}}_{\text{Sample}})/{\text{A}}_{\text{Control}}]\times 100$$

### DPPH radical scavenging method

DPPH radical scavenging potential of *Oxalis corniculata* Linn. in different solvent extracts and formulated soap were determined through a spectrophotometric method using ascorbic acid as a standard. A volume of 0.5 ml of plant extract was mixed properly with 2 ml of 1 mM DPPH (2,2-diphenyl-1-picrylhydrazyl) solution. The absorbance was taken at 517 nm after 1 h incubation at room temperature [34].

$$\text{DPPH radical scavenging rate}\;(\%)=[({\text{Absorbance}}_{\text{Control}})-{\text{Absorbance}}_{\text{Sample}}]/{\text{Absorbance}}_{\text{Control}}\times 100$$

### Agar well diffusion method

This method was used to measure the antibacterial activity of the *Oxalis corniculata* Linn. in different solvent extracts and formulated soap against two Gram-positive bacteria *Staphylococcus aureus* (ATCC25923-0360P) and *Bacillus subtilis* (ATCC11774-0269P), and two Gram-negative bacteria *Escherichia coli* (ATCC35218-0495P) and *Salmonella typhi* (ATCC14028-0363P). The bacterial strains were cultured in 20 ml nutrient broth medium and incubated for a day with 101 revolutions per minute (r.p.m.) (New Brunswick Scientific Excella E24 Incubator Shaker) to enhance their growth. The wells were made with a sterile cork borer after spreading the inoculum over the agar into which plant extracts were poured and incubated at 37 °C for 24 h to obtain zones of growth inhibition. Here, chloramphenicol was used as positive control [35,36].

### Determination of minimum inhibitory concentration (MIC)

The antibacterial potential of the different decoctions of whole plant of *Oxalis corniculata* Linn. was evaluated by microbroth dilution assay in 96-well plates. The desired inoculum of bacterial strains were prepared overnight at 37  °C in Mueller-Hilton agar broth possessing 0.5 McFarland turbidity standard at 600 nm OD (optical density). Then 100 µl Mueller-Hilton agar was added in all the 12 wells of each row. In wells 4–12, 10 µl of varying concentrations (0.04–10 µg ml^−1^) of different decoctions of *Oxalis corniculata* Linn. were added. Additionally, each well received 5 µl of bacterial suspension except the first well which served as negative control. The microtitre plate was allowed to incubate at 37 °C for 24 h and growth was observed by visual inspection. The lowest concentration of the extract at which no visible growth was observed was recorded as the MIC.

### Experimental design and statistical analysis

Every experiment was performed thrice. One-way ANOVA was used to determine the difference in mean values and represented in the form of mean±standard deviation using GraphPad Prism5 software Version 5.03 (San Diego, United States of America). After performing an ANOVA to determine the impact of various wavelengths and the percentage of solvent used on the physical qualities of the soap, the process was optimized using response surface technique. With two independent variables (factors), such as different wavelengths (X_1_) and the percentage of solvent used (X_2_), on the dependent variables (responses), absorbance (Y_1_) was measured. Five centre points were used in thirteen trials. Table 1 lists the two factors together, along with their actual and coded values. The design used five replicates in the centre.

**Table 1.** List of independent variables and their levels

Independent variables	Unit	Symbol	Coded levels				
			-2	-1	0	+1	+2
Wavelength	nm	X_1_	250	300	350	400	450
Percent of solvent	ml	X_2_	50	60	70	80	90

According to lack-of-fit tests, multiple correlation coefficients (R_2_), sequential model sum of squares (SMSS), and lack-of-fit-tests, linear, quadratic, or cubic models for each response variable were chosen. Because there weren't enough points to support a cubic model, it was aliased. With this data, a quadratic model was selected as the most plausible explanation. The following quadratic regression formula was used to fit the experimental response functions of second order:

$$Y_{0}=\beta_{0}+\beta_{1}X_{1}+\beta_{2}X_{2}+\beta_{11}X_{1}^{2}+\beta_{22}X_{2}^{2}+\beta_{12}X_{1}X_{2}$$

Where X_1_ is the different wavelength; X_2_ is the percentage of solvent used; the fitted response value at the centre of the design, point (0,0), is β_0_; the linear regression terms are β_1_ and β_2_; the quadratic regression terms are β_11_ and β_22_; and the cross product regression term is β_12_. The means for the scores obtained for each of the quality attributes of each product were independently calculated after three replications of each study. The software Design Expert Version 7.1.6 (Stat-Ease, Inc., Minneapolis, USA) was used to perform statistical analysis of variance (ANOVA) and multiple regressions in order to fit the equation. Results included factors like predicted model coefficients, a goodness-of-fit test, and regression coefficients.

### High-performance liquid chromatography (HPLC) analysis

Indian Sorrel (*Oxalis corniculata* Linn.) samples were analysed for the detection of bioactive compounds in a C18 reverse phase HPLC column system with dimensions 4.6 mm × 250 mm, particle size 5 µm. The mobile phase consists of 0.1 % trichloroacetic acid in HPLC grade H_2_O and 90 % acetonitrile in a constant gradient. A diluted extract was injected in a volume of 10 µl. The flow rate was 1 ml min^−1^. Total run time was 15 min. The chromatograms were obtained at 254 nm for analysis of hesperidin, quercetin and gallic acid. The identification as well as quantitation of bioactive compound in sample extract were carried out by using the retention time, absorption spectra, peak areas of the respective standard and expressed as micrograms per millilitre (μg ml^−1^) [29,35].

## Results

### Total phenolic content (TPC) and total flavonoid content (TFC)

**Fig. 3.**
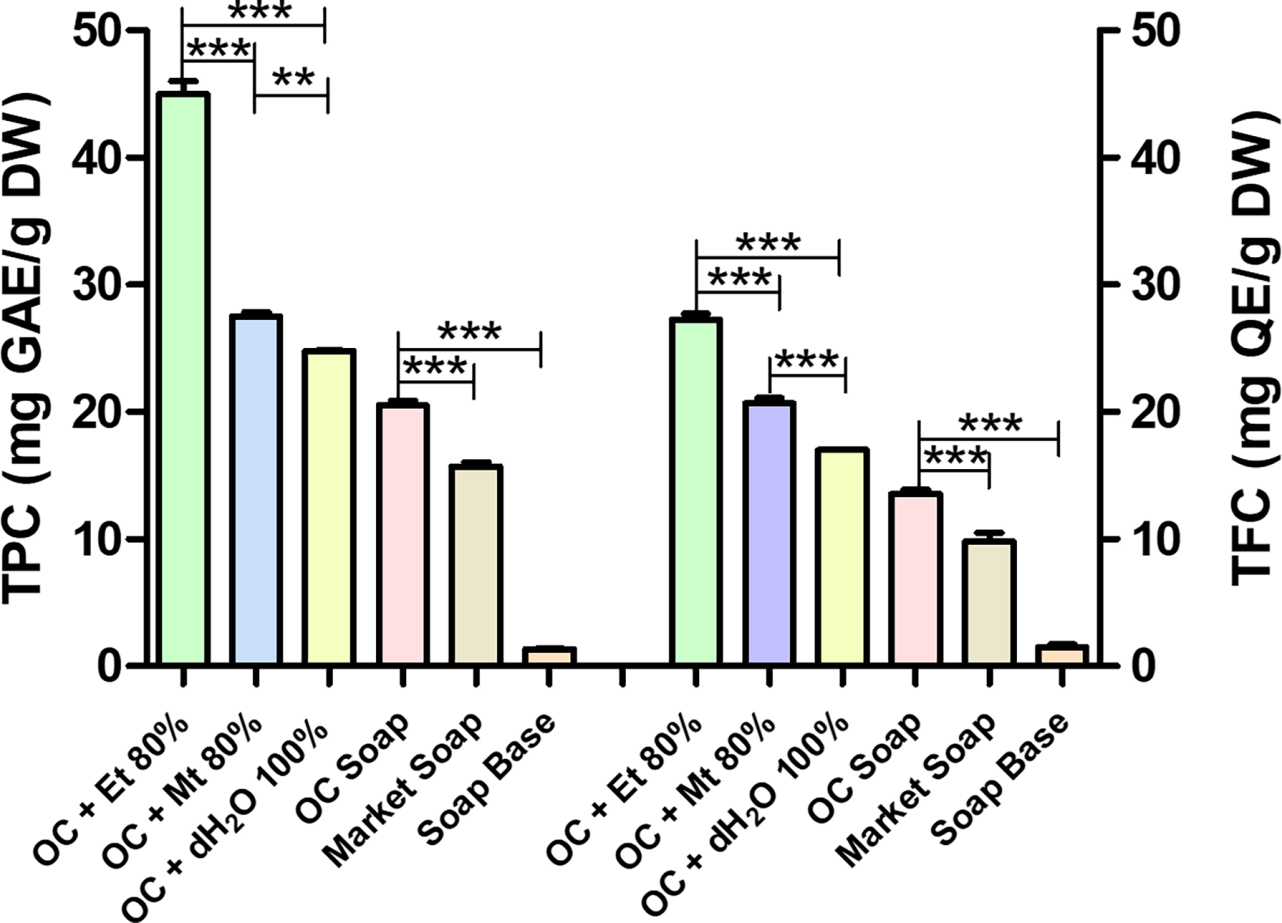
Results of TPC and TFC of *Oxalis corniculata* Linn in different solvent extracts. Bar shows mean±SD with *0.05≥*P*, **0.01≥*P*, ***0.001≥*P*. Abbreviations: OC + Et 80 % - *Oxalis corniculata* Linn + Ethanol 80 %; AA + Et 80 % – Ascorbic Acid + Ethanol 80 %; OC + Mt 80 % - *Oxalis corniculata* Linn + Methanol 80 %; AA + Mt 80 % – Ascorbic Acid + Methanol 80 %; OC + dH_2_O 100 % - *Oxalis corniculata* Linn + Distilled water 100 %; AA + dH_2_O 100 % - Ascorbic Acid + Distilled water 100 %; OC - *Oxalis corniculata* Linn.

The 80 % ethanolic extract of *Oxalis corniculata* Linn. possesses higher TPC (45.025±1.011 mg GAE/g DW) in comparison to its 80 % methanolic (27.520±0.339 mg GAE/g DW) and 100 % distilled water (24.825±0.021 mg GAE/g DW) extracts (Fig. 3). The soap formulated with *Oxalis corniculata* Linn. powder exerts significantly high TPC value (20.505±0.389 mg GAE/g DW) than the market soap (15.710±0.354 mg GAE/g DW) and soap base (1.335±0.120 mg GAE/g DW) (Fig. 3). The TFC value of 80 % ethanolic extract of *Oxalis corniculata* Linn. was 27.250±0.495 mg QE/g DW which greater than the 80 % methanolic and 100 % aqueous extract solution of *Oxalis corniculata* Linn. having TFC values of 20.700±0.424 mg QE/g DW and 17.000±0.000 mg QE/g DW respectively (Fig. 3). In case of formulated soap made up with *Oxalis corniculata* Linn. powder extract*,* the TFC value was 13.550±0.354 mg QE/g DW that was much higher than the market soap (9.800±0.707 mg QE/g DW) and soap base (1.505±0.240 mg QE/g DW) as presented in Fig. 3.

### FRAP assay and TAC assay

**Fig. 4.**
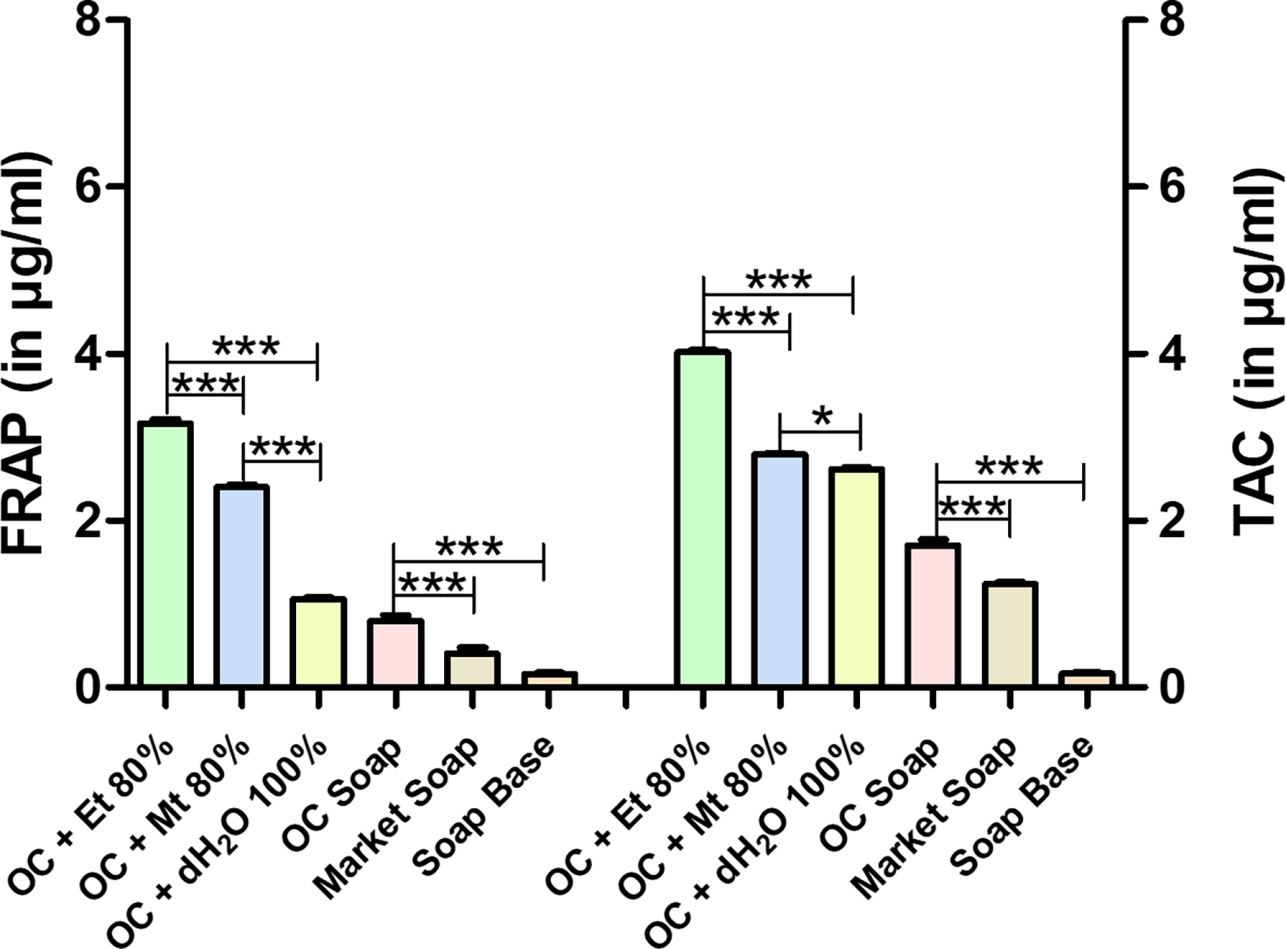
Results of FRAP and TAC of *Oxalis corniculata* Linn in different solvent extracts. Bar shows mean±SD with *0.05≥*P*, **0.01≥*P*, ***0.001≥*P*. Abbreviations: OC + Et 80 % - *Oxalis corniculata* Linn + Ethanol 80 %; AA + Et 80 % – Ascorbic Acid + Ethanol 80 %; OC+Mt 80 % - *Oxalis corniculata* Linn + Methanol 80 %; AA + Mt 80 % – Ascorbic Acid + Methanol 80 %; OC + dH_2_O 100 % - *Oxalis corniculata* Linn + Distilled water 100 %; AA + dH_2_O 100 % - Ascorbic Acid + Distilled water 100 %; OC - *Oxalis corniculata* Linn.

The result of FRAP assay of 80 % ethanolic, 80 % methanolic and 100 % distilled water decoction of whole plant of *Oxalis corniculata* Linn. was represented in Fig. 4. The highest ferric ion reduction potential was found in 80 % ethanolic extract of *Oxalis corniculata* Linn., i.e. 3.160±0.058 µg ml^−1^ followed by 2.394±0.035 µg ml^−1^ of 80 % methanolic extract and 1.058±0.019 µg ml^−1^ of 100 % distilled water extract. The soap formulated with *Oxalis corniculata* Linn. powder extract*,* exerts FRAP value of 0.799±0.066 µg ml^−1^ (Fig. 4). The value of TAC of 80 % methanolic (2.793±0.017 µg ml^−1^) and 100 % distilled water (2.619±0.016 µg ml^−1^) decoction of whole plant of *Oxalis corniculata* Linn. was significantly lower than the 80 % ethanolic extract solution (4.015±0.025 µg ml^−1^) as shown in Fig. 4. The formulated soap exhibits higher TAC value (1.701±0.069 µg ml^−1^) than the market soap (1.240±0.023 µg ml^−1^) and soap base (0.163±0.013 µg ml^−1^) as represented in Fig. 4.

### ABTS assay

**Fig. 5.**
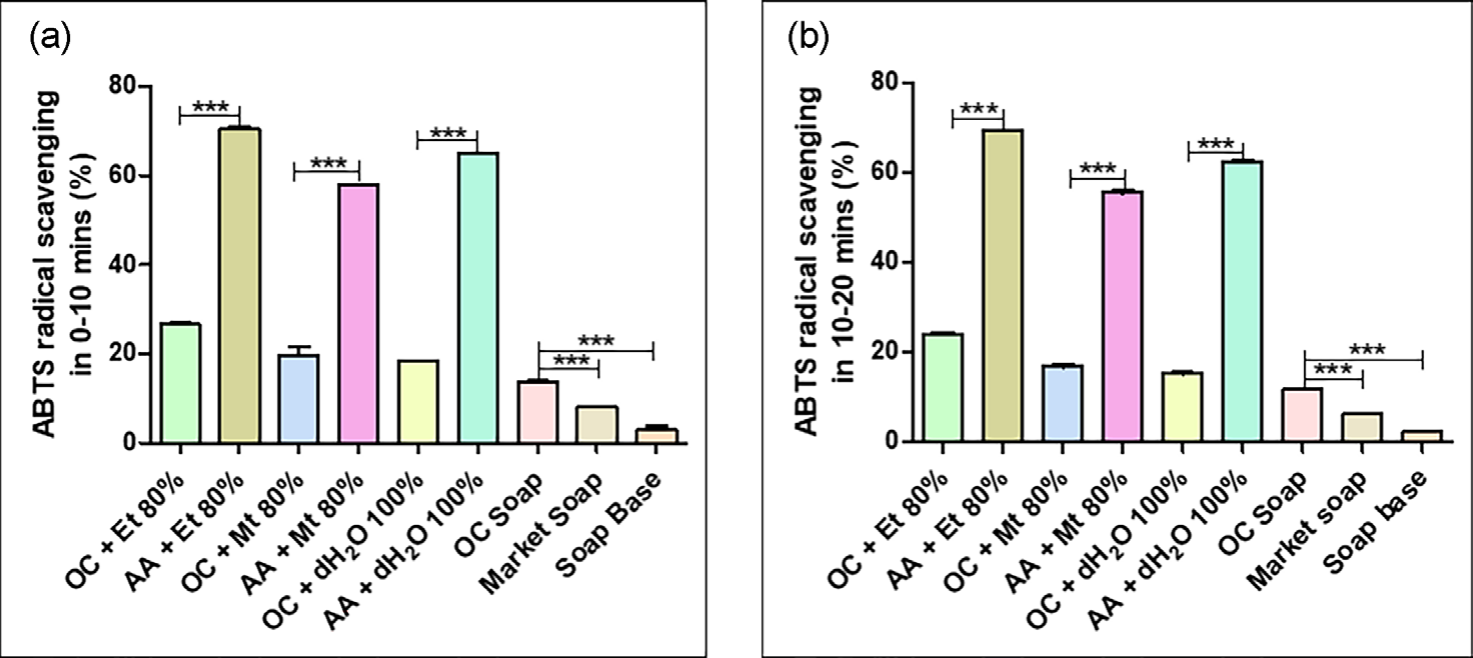
ABTS radical scavenging potential of *Oxalis corniculata* Linn in different solvent extracts in (**a**) 0–10 min and (**b**) 10–20 min. Bar shows mean±SD with *0.05≥*P*, **0.01≥*P*, ***0.001≥*P*. Abbreviations: OC + Et 80 % - *Oxalis corniculata* Linn + Ethanol 80 %; AA + Et 80 % – Ascorbic Acid + Ethanol 80 %; OC + Mt 80 % - *Oxalis corniculata* Linn + Methanol 80 %; AA + Mt 80 % – Ascorbic Acid + Methanol 80 %; OC + dH_2_O 100 % - *Oxalis corniculata* Linn + Distilled water 100 %; AA + dH_2_O 100 % - Ascorbic Acid + Distilled water 100 %; OC - *Oxalis corniculata* Linn.

The results of ABTS assay were depicted in Fig. 5a and b. The 80 % ethanolic decoction of whole plant of *Oxalis corniculata* Linn. exhibits maximum ABTS% inhibition after 10 and 20 min. In the case of 0–10 min, the ABTS radical inhibition potential was 26.583±0.647 % for 80 % ethanolic extract, 19.767±2.331 % for 80 % methanolic extract and 18.414±0.031 % for 100 % distilled water extract solution (Fig. 5a). For 10–20 min the reduced inhibition percent was 24.126±0.151 % for 80 % ethanolic extract, 16.817±0.214 % for 80 % methanolic extract and 15.255±0.091 % for 100 % distilled water extract solution (Fig. 5b). The soap formulated with *Oxalis corniculata* Linn. powder exerts higher ABTS scavenging activity than market soap and soap base at a rate of 13.770±0.382 % and 11.566±0.013 % after 10 and 20 min respectively.

### DPPH assay

**Fig. 6.**
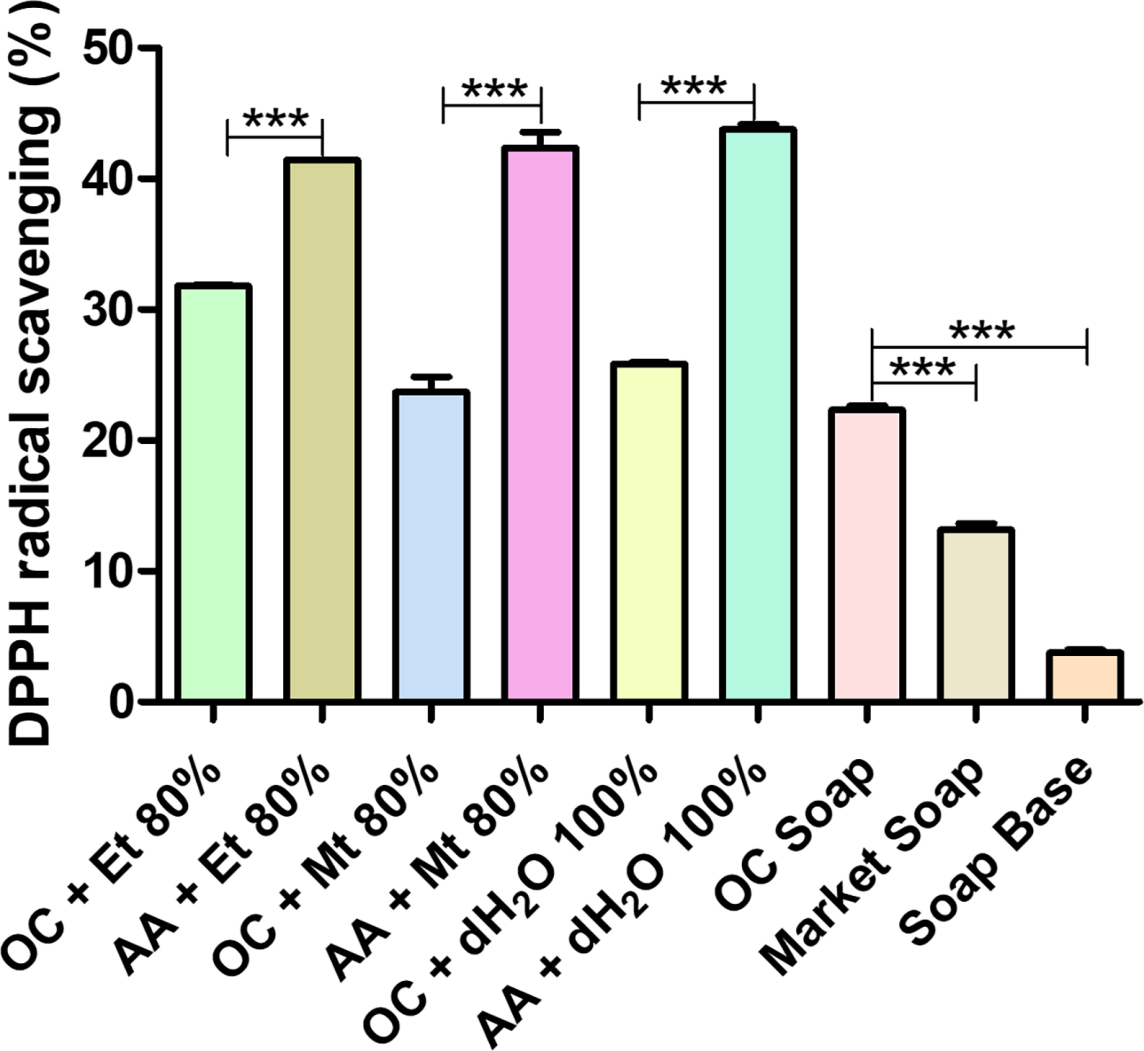
DPPH radical scavenging potential of *Oxalis corniculata* Linn in different solvent extracts. Bar shows mean±SD with *0.05≥*P*, **0.01≥*P*, ***0.001≥*P*. Abbreviations: OC + Et 80 % - *Oxalis corniculata* Linn + Ethanol 80 %; AA + Et 80 % – Ascorbic Acid + Ethanol 80 %; OC + Mt 80 % - *Oxalis corniculata* Linn + Methanol 80 %; AA + Mt 80 % – Ascorbic Acid + Methanol 80 %; OC + dH_2_O 100 % - *Oxalis corniculata* Linn + Distilled water 100 %; AA + dH_2_O 100 % - Ascorbic Acid + Distilled water 100 %; OC - *Oxalis corniculata* Linn.

DPPH assay potential of different decoctions of whole plant of *Oxalis corniculata* Linn. was depicted in Fig. 6. Among these three, the 80 % ethanolic solution showed highest DPPH inhibition activity, i.e. 31.805±0.127 % followed by 100 % distilled water (25.854 % ± 0.198 %) and 80 % methanolic solution (23.675 % ± 1.642 %). The formulated soap possesses good DPPH inhibition potential, i.e. 22.325±0.460 % which is higher than that of market soap (13.140±0.721 %) and soap base (3.770±0.297 %).

### Antibacterial activity assay

**Fig. 7.**
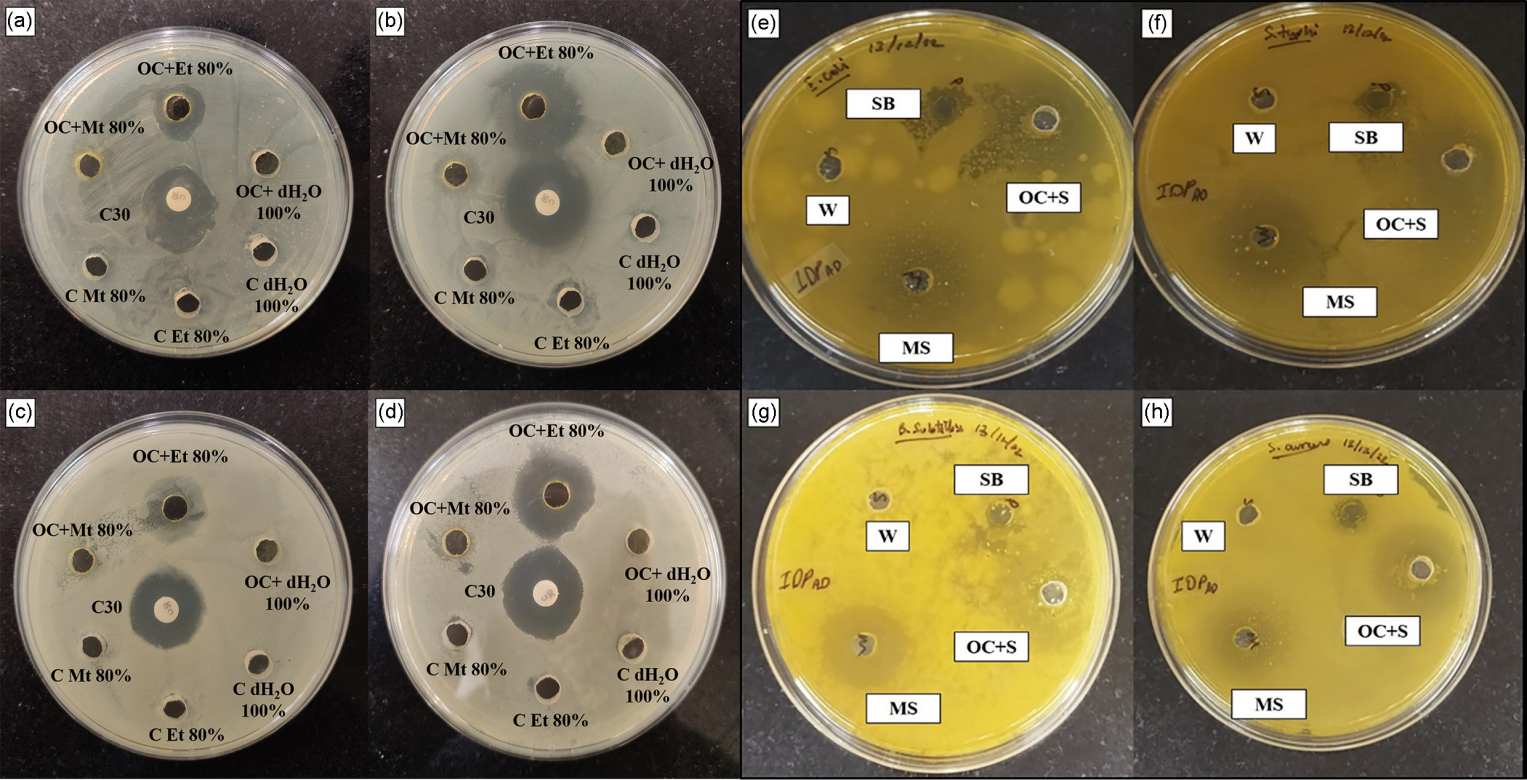
Antibacterial activity of *O. corniculata* Linn in different solvent and formulated soap against bacterial strains (**a, e**) *E. coli*, (**b, f**) *S. typhi*, (**c, g**) *B. subtilis*, (**d, h**) *S. aureus*. Abbreviations: OC + Mt 80 % - *Oxalis corniculata* Linn + Methanol 80 %; OC + Et 80 % - *Oxalis corniculata* Linn + Ethanol 80 %; OC + dH_2_O 100 % - *Oxalis corniculata* Linn + Distilled water 100 %; C30 - Chloramphenicol C30; C Mt 80 % - Control Methanol 80 %; C Et 80 % - Control Ethanol 80 %; C dH_2_O 100 % - Control Distilled water 100 %; OC + S - Formulated Soap; MS - Market soap; SB - Soap Base; W - Water.

The 80 % methanolic and 80 % ethanolic decoctions of whole plant of Indian Sorrel portray significant antibacterial properties against four bacterial strains (Fig. 7). Here, chloramphenicol (C30) showed highest antibacterial activity by serving as a positive control. Among all extracts, the 80 % ethanolic decoctions showed highest zone of inhibition with diameter ranges from 14.650±0.141 mm for *Escherichia coli*, 23.250±0.354 mm for *Salmonella typhi*, 17.240±0.014 mm for *Bacillus subtilis,* and 20.500±0.707 mm for *Staphylococcus aureus* (Table 2). The formulated soap also exhibits greater bacterial growth inhibitory effect (8.730±0.028 mm for *Escherichia coli,* 12.735±0.728 mm for *Salmonella typhi*, 9.225±0.035 mm for *Bacillus subtilis,* and 12.225±0.035 mm for *Staphylococcus aureus*) in comparison to the market soap (5.225±0.035 mm for *E. coli,* 8.720±0.042 mm for *S. typhi*, 6.735±0.021 mm for *Bacillus subtilis,* and 8.495±0.007 mm for *Staphylococcus aureus*) (Table 2).

**Table 2.** Table showing the zone of growth inhibition (in mm) by the *Oxalis corniculata* Linn in different solvent extracts and formulated soap against different bacterial strains. The values are shown as mean±SD (*n*=3) with *0.05≥*P*, **0.01≥*P*, ***0.001≥*P*. Here the inhibition of bacterial strains are compared with chloramphenicol C30

	Zone of growth inhibition (mm)			
Sample	*Escherichia coli*	*Salmonella Typhi*	*Bacillus subtillis*	*Staphylococcus aureus*
OC + Et 80 %	14.650±0.141***	23.250±0.354^ns^	17.240±0.014***	20.500±0.707***
OC + Mt 80 %	3.725±0.035***	13.900±0.141***	4.400±0.141***	3.730±0.028***
OC + dH_2_O 100 %	0.000±0.000***	0.000±0.000***	0.000±0.000***	0.000±0.000***
Chloramphenicol C30	20.125±0.177	23.350±0.212	18.850±0.212	22.225±0.035
C Et 80 %	3.715±0.049***	11.400±0.141***	2.300±0.283***	3.245±0.007***
C Mt 80 %	2.245±0.007***	7.725±0.035***	3.350±0.212***	4.650±0.141***
C dH_2_O 100 %	0.000±0.000***	0.000±0.000***	0.000±0.000***	0.000±0.000***
OC soap	8.730±0.028***	12.735±0.728***	9.225±0.035***	12.225±0.035***
Market soap	5.225±0.035***	8.720±0.042***	6.735±0.021***	8.495±0.007***
Soap base	0.000±0.000***	0.000±0.000***	0.000±0.000***	0.000±0.000***
Water	0.000±0.000^***^	0.000±0.000***	0.000±0.000***	0.000±0.000***

### Minimum inhibitory concentration

**Fig. 8.**
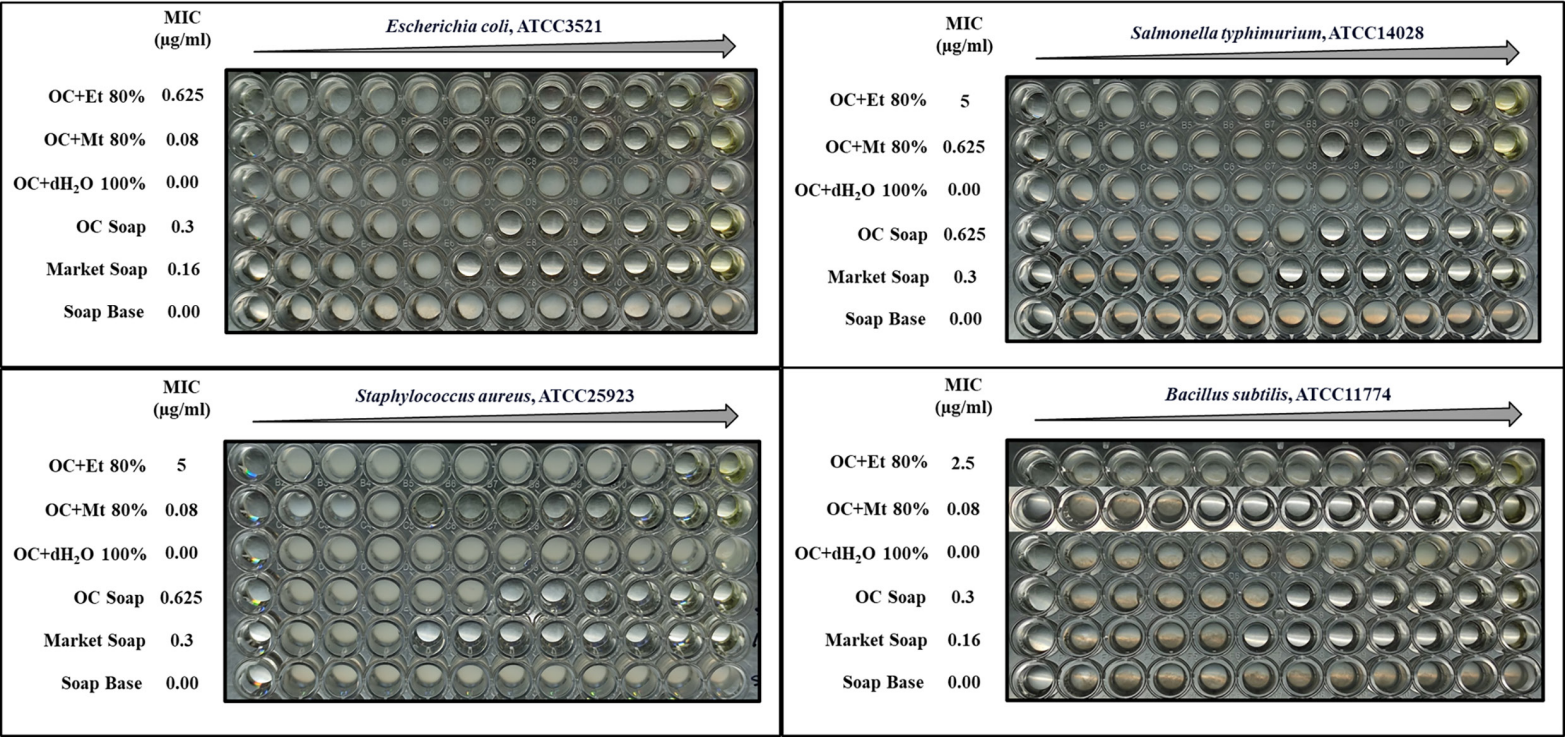
MIC values of different decoctions of *Oxalis corniculata* Linn. against *Escherichia coli, Salmonella typhimurium, Staphylococcus aureus and Bacillus subtillis*. Abbreviations: OC + Et 80 % - *Oxalis corniculata* Linn + Ethanol 80 %; OC + Mt 80 % - *Oxalis corniculata* Linn + Methanol 80 %; OC + dH_2_O 100 % - *Oxalis corniculata* Linn + Distilled water 100 %; OC - *Oxalis corniculata* Linn.

The effectiveness of different decoctions of whole plant of *Oxalis corniculata* Linn. in different bacterial strains was evaluated by determining the minimum inhibitory concentration (MIC) (Fig. 8). Among all different decoctions tested, the 80 % ethanolic decoction always exhibits the strongest antimicrobial potential. The MIC of two Gram-negative bacterial strains, i.e. *Escherichia coli* and *Salmonella typhimurium* were 0.625 µg ml^−1^ and 5 µg ml^−1^ respectively. The MIC of *Bacillus subtillis* was 2.5 µg ml^−1^ and *Staphylococcus aureus* was 5 µg ml^−1^. The 80 % methanolic decoctions of whole plant of Indian Sorrel was also effective against *Escherichia coli, Salmonella typhimurium, Bacillus subtillis,* and *Staphylococcus aureus* with MIC values of 0.08 µg ml^−1^, 0.625 µg ml^−1^, 0.08 µg ml^−1^, and 0.08 µg ml^−1^, respectively. The formulated soap exerts greater MIC values (0.3 µg ml^−1^ against both *Escherichia coli* and *Bacillus subtillis,* 0.625 µg ml^−1^ against *Salmonella typhimurium* and *Staphylococcus aureus*) as compared to the market soap.

### Effects of different wavelengths and percent of solvent used on absorbance

**Fig. 9.**
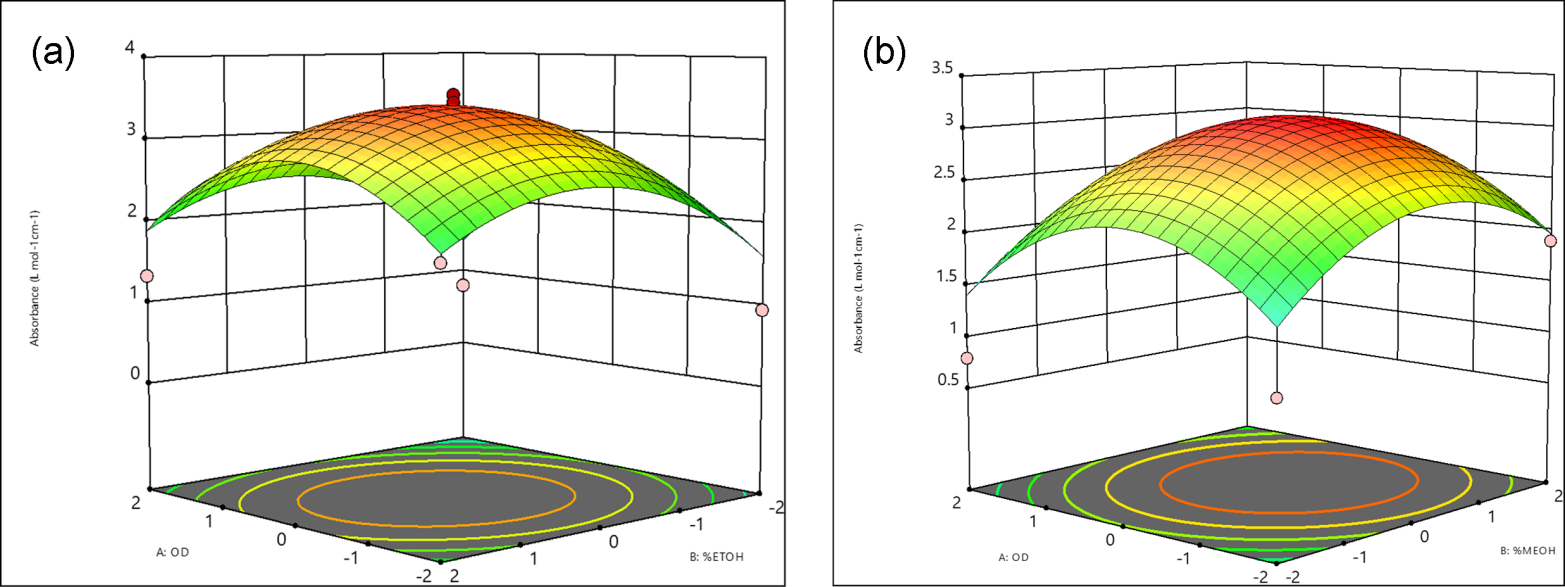
The 3D RSM plots to visualize the interaction effects of the variables in (**a)** 80 % ethanol and (**b)** 80 % methanol extract.

ANOVA for the RSM quadratic model 80 % ethanolic and 80 % methanolic decoction was given in Tables3  4 respectively. The regression coefficient value of 80 % ethanolic and 80 % methanolic decoction of this model were 0.75 and 0.70 respectively. More fitted data are shared by 80 % methanolic decoction in the response data used to fit the model. The combined impact of various wavelengths and the percentage of solvent used on absorbance is shown in Fig. 9.

**Table 3.** ANOVA for the RSM quadratic model 80 % ethanolic decoction

	Sum of squares	Degree of freedom	Mean square	F-value	*P*-value
**Regression (model**)	18.41	8	2.30	6.67	**<0.0005**
**Residual**	5.87	17	0.3456		
**Lack of fit**	5.58	9	0.62	17.37	**0.0002**
**Pure error**	0.2855	8	0.0357		
**Corrected total**	24.28	25			
**R^2^=0.7584; adjusted R^2^=0.6447; CV=25.21 %**					

**Table 4.** ANOVA for the RSM quadratic model 80 % methanolic decoction

	Sum of squares	Degree of freedom	Mean square	F-value	*P*-value
**Regression (model**)	12.48	8	1.56	5.05	**<0.0025**
**Residual**	5.25	17	0.3088		
**Lack of fit**	5.25	9	0.5833	9.721E+05	**0.0001**
**Pure error**	4.800E-06	8	6.00E-07		
**Corrected total**	17.73	25			
**R^2^=0.7039; adjusted R^2^=0.5646; CV=25.36 %**					

### Detection of bioactive compounds

**Fig. 10.**
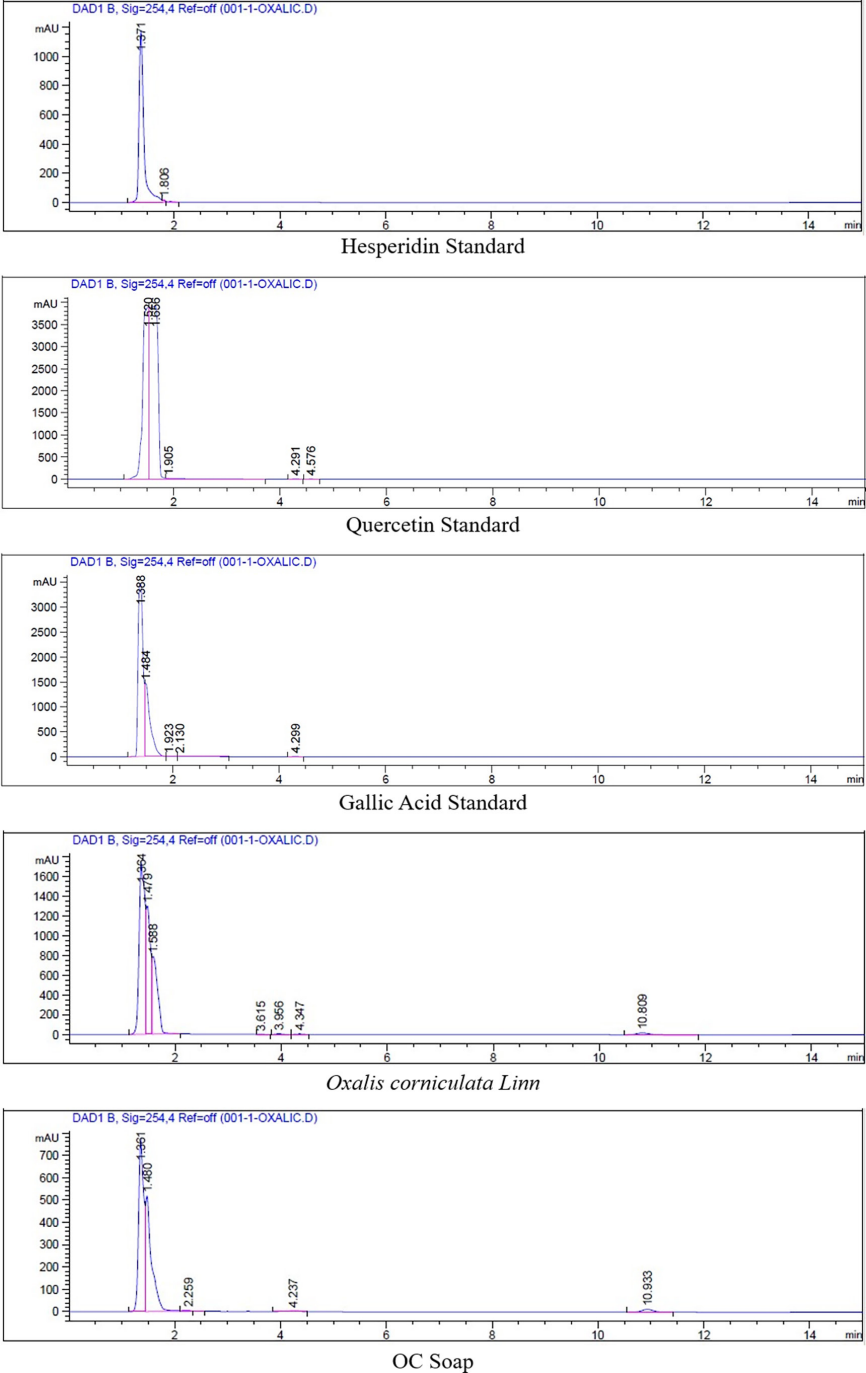
HPLC analysis.

In order to ascertain whether or not any bioactive compound was present in samples such as crude and product, we analysed the samples by HPLC. The peak at 1.371 min was identified as that of hesperidin. Sample crude and product also showed a peak at the same retention time, i.e. 1.479 and 1.480 respectively at 254 nm which corresponded to concentration of hesperidin 0.0933 mg ml^−1^ in crude *Oxalis corniculata* Linn. and 0.0542 mg ml^−1^ in OC soap as shown in figure. Similarly, a peak at 1.520 min was identified for quercetin. Sample crude and product showed a peak at 254 nm at the same retention time, i.e. 1.588 and 1.480 respectively as the quercetin standard which corresponded to concentration of quercetin 0.1968 mg ml^−1^ in crude *Oxalis corniculata* Linn. and 0.1462 mg ml^−1^ in OC soap as shown in Figure. Likewise, the peak at 1.388 min was identified as that of gallic acid. Sample crude and product showed a peak at 254 nm at the same retention time, i.e. 1.364 and 1.361 respectively as the gallic acid standard which corresponded to concentration of gallic acid 0.0917 mg ml^−1^ in crude *Oxalis corniculata* Linn. and 0.0682 mg ml^−1^ in OC soap as shown in Fig. 10.

## Discussion

In the past several decades, a huge number of reports have explored the antioxidant, cardioprotective, neuroprotective, anti-diabetic, antihypertensive, anti-inflammatory, and anticarcinogenic potential of different bioactive compounds isolated from low-cost agricultural resources [26,37]. For instance, apple peels, grape skins, avocado seeds, etc. possess approximately 15 % higher polyphenolic compounds as compared to the fruit pulp. However, due to lack of proper knowledge and experimental evidence a huge number of agricultural resources are mismanaged in the form of agro wastes despite possessing beneficial health effects. The herbaceous plant *Oxalis corniculata* Linn. (Oxalidaceae) is commonly used as an antibacterial, refrigerant, diaphoretic, and wound-healing agent in traditional medical practises such as Ayurveda, Unani, and Siddha [3, 10]. It is enriched with several bioactive compounds including essential fatty acids, vitamin C, carotenoids, flavonoids, phytosterol, phenol, tannins, etc. [20,38]. Although several studies have been conducted to explore the potential of the Indian Sorrel leaf extract, the scavenging activity as well as antimicrobial efficiency of *Oxalis corniculata* Linn. leaf extract in different decoctions were investigated in this study due to the importance and necessity of formulating a therapeutic soap.

In the present study, two free radical scavenging activity assays (DPPH, ABTS) were used to ascertain the antioxidant potential. The results obtained from these tests were almost consistent, i.e. the 80 % ethanolic extract exhibited maximum radical scavenging activity as well as antioxidant properties which may be due to its greater TPC and TFC. In one earlier study, it was reported that oral administration of ethanolic extract of *Oxalis corniculata* in Wistar rats attenuated the oxidative stress mediated hepatotoxic effects of thioacetamide. The antioxidant constituents such as flavonoids and vitamin C present in ethanolic extract of *Oxalis corniculata* were mainly responsible for offering this hepatoprotective activity [39]. In this research, among the 80 % ethanolic, 80 % methanolic and 100 % distilled water decoctions of *Oxalis corniculata* Linn. plant extracts, the 80 % ethanolic decoction of *Oxalis corniculata* Linn. plant showed significantly higher bacterial growth inhibitory properties in agar well diffusion assays and MIC assays followed by the 80 % methanolic decoction against four different bacterial strains. This result was similar to a study conducted by [40] which revealed that leaves of *Oxalis corniculata* possess highest antibacterial potential due to its higher flavonoid and phenolic contents than the seeds [40,41]. Another report [42], found that methanolic and ethanolic extracts of *Oxalis corniculata* Linn. plant showed significant antibacterial activity against phytopathogenic (e.g. *Xanthomonas pathovars*) as well as human pathogenic bacteria with the help of its phenolic constituents [42]. Phytochemical analysis revealed that in ethanolic and methanolic extracts of *Oxalis corniculata,* the presence of phenolic compounds, flavonoids, carbohydrates, amino acids, proteins, volatile oil, citric acid, tartaric acid, fibres, and tannins are higher [42,43]. The HPLC analysis performed in this study had confirmed the presence of hesperidin, quercetin and gallic acid in the crude sample and OC soap which are responsible for governing the antioxidant and antibacterial activities. Therefore, according to the results obtained in this research, the 80 % ethanolic extract of *Oxalis corniculata* Linn. was selected to be used to achieve good antioxidant and antibacterial activity in herbal soap formulation.

Presently, although there is a wide range of standard soaps are available in the market, however it has some drawbacks such as its preservatives content, petroleum based lathering agents such as synthetic fragrances, harsh dyes or colouring agents, etc. which over long-time usage may lead to harmful effects on the skin. In contrast to this, the organic soaps which are made up of natural ingredients, herbs or any sort Ayurvedic compound are cruelty-free and animal-friendly. The pH level of organic products is between 9 and 10 (much basic in nature). It means that it is gentler on your skin, and it makes the skin less prone to irritation. Organic soaps are loaded with glycerine, also known as glycerol, which is also a natural alcohol and water attractor that is found in soaps. Beside from its cleansing properties, it is considered the best moisturizing agent for the human skin, and has been in use for centuries. Organic products use natural antibacterial agents such as essential oils (like peppermint, tea-tree, lavender, and lemongrass) for cleansing, moisturizing and adding fragrance. Therefore, nowadays organic soaps are gaining more preference over the markedly available soaps [44]. These include soaps manufactured with dried leaves of basil, neem, acalypha, aloe vera, and hibiscus flowers by stream extraction process. In this study, an organic soap was formulated with *Oxalis corniculata* Linn. which offered significantly higher phenolic, flavonoid content, antioxidant and antibacterial potential in comparison to the market soap, which may be due to the enrichment of different beneficial bioactive compounds in the *Oxalis corniculata* Linn. extract. In one similar study done earlier, a bath soap formulated with different bioactive herbal plant extracts was compared with market soap. The herbal soap besides having good appearance, colour, and fragrance, was also found to possess good antioxidant and antibacterial properties due to its constituent bioactive components [45]. A soap formulated with seed coat of *Borassus flabellifer* and rhizome of *Curcuma zedoaria* displayed good antimicrobial activity due to the bioactive compounds present in the herbal extract as determined by GC MS analysis [46]. The liquid medicinal soap prepared from pomegranate (*Punica granatum*) leaf extracts retained both the actual antioxidant and antibacterial activity of the crude plant extract, whereas the solid soap possesses only the antioxidant activity [47]. Utilization of this kind of antioxidant as well as antibacterial active soap in our daily lifestyle can improve the quality of our skin health and also counteract the rising skin disease death cases.

## Abbreviations

ABTS: 2,2-azino-bis-3-ethylbenzothiazoline-6-sulphonic acid
ANOVA: analysis of variance
DPPH: 2,2-diphenyl-1-picrylhydrazyl
FRAP: ferric reducing antioxidant power assay
HPLC: high performance liquid chromatography
RSM: response surface methodology
TAC: total antioxidant assay

## References

[1] Gokmen V (2015). Acrylamide in Food: Analysis, Content and Potential Health Effects.

[2] Bernhoft AJ (2010). Bioactive Compounds in Plants-Benefits and Risks for Man and Animals.

[3] Taranalli AD, Tipare SV, Kumar S, Torgal SS (2004). Wound healing activity of *Oxalis corniculata* whole plant extract in rats. Indian J Pharm Sci.

[4] Kumar H, Bhardwaj K, Sharma R, Nepovimova E, Kuča K (2020). Fruit and vegetable peels: utilization of high value horticultural waste in novel industrial applications. Molecules.

[5] Silva Dias J (2010). World importance, marketing and trading of vegetables.

[6] Hussain A, Kausar T, Din A, Murtaza MA, Jamil MA (2021). Determination of total phenolic, flavonoid, carotenoid, and mineral contents in peel, flesh, and seeds of pumpkin (*Cucurbita maxima*). J Food Process Preserv.

[7] Singla M, Singh A, Sit N (2023). Effect of microwave and enzymatic pretreatment and type of solvent on kinetics of ultrasound assisted extraction of bioactive compounds from ripe papaya peel. J Food Process Eng.

[8] Sirijan M, Pipattanawong N, Saeng-on B, Chaiprasart P (2020). Anthocyanin content, bioactive compounds and physico-chemical characteristics of potential new strawberry cultivars rich in-anthocyanins. J Berry Res.

[9] Szymanowska U, Baraniak B (2019). Antioxidant and potentially anti-inflammatory activity of anthocyanin fractions from pomace obtained from enzymatically treated raspberries. Antioxidants.

[10] Mohan SM, Pandey B, Deshpande B, Chandrakar V (2015). Antibacterial activity of plant extract of *Oxalis corniculata*. Indian J Life Sci.

[11] Alparslan Y, Baygar T (2017). Effect of chitosan film coating combined with orange peel essential oil on the shelf life of deepwater pink shrimp. Food Bioprocess Technol.

[12] Yang F, Wang Y, Zhao H (2019). Quality enhancement of fermented vegetable juice by probiotic through fermented yam juice using *Saccharomyces cerevisiae*. Food Sci Technol.

[13] Sobota A, Wirkijowska A, Zarzycki P (2020). Application of vegetable concentrates and powders in coloured pasta production. Int J of Food Sci Tech.

[14] Das P, Kumar K, Nambiraj A, Awasthi R, Dua K (2019). Antibacterial and in vitro growth inhibition study of struvite urinary stones using *Oxalis corniculata* Linn. Leaf extract and its biofabricated silver nanoparticles. Recent Pat Drug Deliv Formul.

[15] Salas-Millán JÁ, Aznar A, Conesa E, Conesa-Bueno A, Aguayo E (2022). Functional food obtained from fermentation of broccoli by-products (stalk): metagenomics profile and glucosinolate and phenolic compounds characterization by LC-ESI-QqQ-MS/MS. LWT.

[16] Lemes AC, Álvares GT, Egea MB, Brandelli A, Kalil SJ (2016). Simultaneous production of proteases and antioxidant compounds from agro-industrial by-products. Bioresour Technol.

[17] Durgawale PP, Hendre AS, Phatak RS (2015). GC/MS characterization, antioxidant and free radical scavenging capacities of methanolic extract of *Oxalis corniculata* LINN: an ayurvedic herb. Rasayan J Chem.

[18] Dhua S, Kumar K, Sharanagat VS, Nema PK (2022). Bioactive compounds and its optimization from food waste: review on novel extraction techniques. NFS.

[19] Arah IK, Ahorbo GK, Anku EK, Kumah EK, Amaglo H (2016). Postharvest handling practices and treatment methods for tomato handlers in developing countries: a mini review. Adv Agric.

[20] Ahmed D, Zara S, Baig H (2013). In vitro analysis of antioxidant activities of *Oxalis corniculata* Linn. fractions in various solvents. Afr J Tradit Complement Altern Med.

[21] Ishangulyyev R, Kim S, Lee SH (2019). Understanding food loss and waste-why are we losing and wasting food?. Foods.

[22] Sadh PK, Duhan S, Duhan JS (2018). Agro-industrial wastes and their utilization using solid state fermentation: a review. Bioresour Bioprocess.

[23] Irmak S (2017). Biomass Volume Estimation and Valorization for Energy.

[24] Gorinstein S, Martín-Belloso O, Park Y-S, Haruenkit R, Lojek A (2001). Comparison of some biochemical characteristics of different citrus fruits. Food Chem.

[25] Banerjee J, Singh R, Vijayaraghavan R, MacFarlane D, Patti AF (2017). Bioactives from fruit processing wastes: green approaches to valuable chemicals. Food Chem.

[26] Lemes AC, Egea MB, de Oliveira Filho JG, Gautério GV, Ribeiro BD (2021). Biological approaches for extraction of bioactive compounds from agro-industrial by-products: a review. Front Bioeng Biotechnol.

[27] Mathur R, Vijayvergia R (2017). Determination of total flavonoid and phenol content in *Mimusops elengi* Linn. Int J Pharm Sci Res.

[28] Shirazi OU, Khattak MM, Shukri NA (2014). Determination of total phenolic, flavonoid content and free radical scavenging activities of common herbs and spices. J Pharmacogn Phytochem.

[29] Torres-León C, Rojas R, Contreras-Esquivel JC, Serna-Cock L, Belmares-Cerda RE (2016). Mango seed: functional and nutritional properties. Trends Food Sci Technol.

[30] Vijayalakshmi M, Ruckmani K (2016). Ferric reducing anti-oxidant power assay in plant extract. Bangladesh J Pharmacol.

[31] Re R, Pellegrini N, Proteggente A, Pannala A, Yang M (1999). Antioxidant activity applying an improved ABTS radical cation decolorization assay. Free Radic Biol Med.

[32] Ballesteros-Vivas D, Álvarez-Rivera G, Morantes SJ, Sánchez-Camargo ADP, Ibáñez E (2019). An integrated approach for the valorization of mango seed kernel: efficient extraction solvent selection, phytochemical profiling and antiproliferative activity assessment. Food Res Int.

[33] Proestos C, Lytoudi K, Mavromelanidou OK, Zoumpoulakis P, Sinanoglou VJ (2013). Antioxidant capacity of selected plant extracts and their essential oils. Antioxidants.

[34] Fanta Yadang SA, Taiwe Sotoing G, Ngatcha Zouakeu KS, Khan MA, Agbor GA (2019). Quantification of bioactive compounds and evaluation of the antioxidant activity of *Carissa edulis* Valh (Apocynaceae) leaves. Sci World J.

[35] Mathivanan D, Suseem SR (2016). Chemical and biological evaluation of *Andrographis echioides* leaf extracts collected from the Vellore district in Tamil Nadu, India. Pac Sci Rev A Nat Sci Eng.

[36] Naqvi SAZ, Irfan A, Zahoor AF, Zafar M, Maria A (2020). Determination of antimicrobial and antioxidant potential of agro-waste peels. An Acad Bras Cienc.

[37] Lemes AC, Paula L, Oliveira Filho J, Prado D, Medronha GA (2020). Bioactive peptides with antihypertensive property obtained from agro-industrial byproducts-mini review. Austin J Nutr Metab.

[38] Das P, Kumar K, Nambiraj A, Awasthi R, Dua K (2019). Antibacterial and in vitro growth inhibition study of struvite urinary stones using *Oxalis corniculata* Linn. Leaf extract and its biofabricated silver nanoparticles. Recent Pat Drug Deliv Formul.

[39] Das K, Kathiriya AK, P KE, K BM, Einstein JW (2012). Evaluation of hepatoprotective activity of aqueous and ethanolic extract of *Oxalis corniculata* against intoxication of thioacetamide induced rats. Rev bras farmacogn.

[40] Kaur S, Kaur G, Singh J (2017). Phytochemical screening and biological potential of methanolic extract of *Oxalis corniculata* using different parts of plant. Res J Chem Sci.

[41] Golbarg H, Mehdipour Moghaddam MJ (2021). Antibacterial potency of medicinal plants including *Artemisia annua* and *Oxalis corniculata* against multi-drug resistance *E. coli*. BioMed Res Int.

[42] Raghavendra MP, Satish S, Raveesha KA (2006). Phytochemical analysis and antibacterial activity of *Oxalis Corniculata*; a known medicinal plant. My Sci.

[43] Ibrahim M, Hussain I, Imran M, Hussain N, Hussain A (2013). Corniculatin A, a new flavonoidal glucoside from *Oxalis corniculata*. Revista Brasileira de Farmacognosia.

[44] Indirani C, Meenambika K, Indhumathy D, Kavinkumar VS (2022). Preparation of soap using a steam extraction process from leaves of *Azadirachta indica*, *Ocimum basilicum*, *Hibiscus-rosa-sinensis* flowers, *Acalypha indica*, and *Aloe barbadensis* Leaflets. IOP Conf Ser: Earth Environ Sci.

[45] Sharma S, Pradhan S, Pandit B, Mohanty JP (2022). Formulation and evaluation of herbal soap taking different bioactive plants by cold saponification method. Int J Curr Pharm Sci.

[46] Nisha PD, Kumar UD (2021). Formulation, development and characterization of herbal soap using *Borassus flabellifer* and *Curcuma zedoaria*. IJPSRR.

[47] Wijetunge WMANK, Perera BGK (2016). Preparation of medicinal soap products using the leaf extracts of Punica Granatum (pomegranate). Int J Pharmacy Biol Sci.

